# Sorption-Enhanced Methanation Using CaO- and Ni-Based Catalysts as Functional Materials

**DOI:** 10.1021/acs.energyfuels.4c04828

**Published:** 2025-02-25

**Authors:** Yusbeli C. García, Gemma Grasa, Isabel Martínez

**Affiliations:** Environmental Research Group, Instituto de Carboquímica (Spanish National Research Council, ICB-CSIC), Miguel Luesma Castán 4, 50018 Zaragoza, Spain

## Abstract

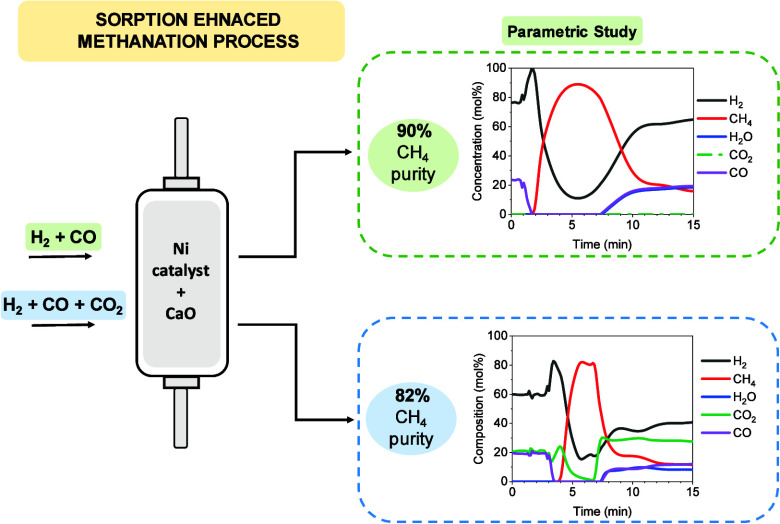

In this work, a detailed parametric study of sorption-enhanced methanation (SEM) to produce synthetic natural gas was carried out. A commercial Ni-based catalyst and CaO as a H_2_O sorbent were used as functional materials. The operational parameters studied included the reaction temperature, H_2_/CO module in the feed gas, CO space velocity, and sorbent/catalyst mass ratio. High CH_4_ purity, close to 90%, was obtained at 275 °C, utilizing a module with H_2_/CO = 3 as the feed gas, 0.8 kg_CO_/kg_cat_ h, and a CaO/catalyst mass ratio of 1. Under these operational conditions, a cycle stability test was performed, demonstrating good reproducibility over 14 cycles. SEM performance was also analyzed using a sintered CaO sorbent. The study demonstrated that the reactivation of the material resulted in similar effectiveness to using calcined CaO. Finally, the process was studied utilizing a H_2_/CO/CO_2_ mixture as the feed gas, obtaining 80% CH_4_ purity in the product gas.

## 1 Introduction

Nowadays, traditional fossil fuels remain as the main demanded primary energy source.^1^ The CO_2_ emissions related to the exploitation of fossil fuels have given rise to concerns about global warming. The current agreements and measures to be adopted focus on the production of renewable energy and the synthesis of alternative fuels.^2^ In this context, synthetic natural gas (SNG) has emerged as an alternative source of methane to natural gas. The production of SNG from biomass gasification has attracted increasing attention due to its potential for the sustainable production of energy.^3^ The produced SNG can be injected in the existing natural gas infrastructure without any limitation, replacing fossil natural gas and its use.^4^ The SNG can serve as a long-term energy storage solution, utilizing excess renewable energy and ensuring a continuous and stable energy supply. In accordance with Directive 2003/55/CE, the European transmission and distribution companies have established national specifications for the injection of SNG into the natural gas infrastructure.^5^ The SNG quality varies according to national regulations but generally requires more than 80% CH_4_ by volume.^6^ In Spain, the minimum CH_4_ content in SNG is established at 95% and the H_2_ concentration is limited to 5% by volume, according to Real Decreto 376/2022.^7^

SNG production is based on methanation reactions, in which the carbon oxides (CO and CO_2_) and H_2_ are converted to CH_4_ and H_2_O. Sabatier and Senderens first explored the catalytic CO and CO_2_ methanation according to eqs 1 and 2, respectively.^8^ Due to thermodynamic limitations, high pressures and low temperatures are needed to reach a high conversion of CO and CO_2_ to CH_4_.
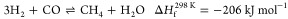
1
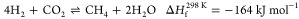
2

The TREMP process is one of the most widely recognized commercial methanation processes. It is used for the production of SNG from synthesis gas (i.e., a mixture of H_2_, CO, and CO_2_). The TREMP process is based on three adiabatic fixed-bed methanation reactors with a recycle stream and intermediate cooling steps. High operating pressures (i.e., up to 30 bar) and temperatures in the range of 250–700 °C are achieved in the first methanation reactor.^6,9^ Such a high temperature reached in the process allows the recovery of heat at high temperatures by means of superheated steam production, which maximizes the efficiency of the process. Due to the significant kinetic limitation of the methanation reactions, a catalyst is required to achieve remarkable rates and selectivities. Transition metals, such as Ni, Fe, and Co, are widely used as CO/CO_2_ methanation catalysts for industrial purposes.^3,10^ In particular, Ni-based catalysts are the most promising active metal for industrial applications due to their high selectivity, high CO/CO_2_ conversion, low price, and availability.^11^ Recognized commercial methanation processes, such as TREMP, achieve complete CO conversion, 95.8% CO_2_ conversion, and 98.2% yield to methane using Ni-based catalysts in the process.^6^

In recent years, many research groups have focused on technologies for CH_4_ production obtained from renewable energy sources (i.e., SNG obtained from biomass gasification and renewable hydrogen). Innovative concepts such as sorption-enhanced methanation (SEM) have been proposed for SNG production. In the SEM process, water produced by the methanation reactions (according to eqs 1 and 2) is continuously removed by a sorbent. This sorption of water promotes equilibrium toward CH_4_ production, reducing secondary reactions and allowing the operation under less stringent conditions of pressure and temperature.^12,13^ Borgschulte et al. first demonstrated this concept using a bifunctional material based on Ni-supported zeolite 5A.^12^ The results showed 100% yield to CH_4_ for the sorption-enhanced CO_2_ methanation reaction under 1.2 bar and temperatures below 300 °C. Bifunctional materials consisting of Ni catalysts supported on zeolite (i.e., zeolites 5A, 13X, and L) have also been investigated by other authors.^14,15^ Wei et al. reported complete CO_2_ conversion and 100% CH_4_ selectivity at 180–300 °C using a catalyst/zeolite bifunctional material.^14^ Delmelle et al. obtained pure CH_4_ with a bifunctional material at atmospheric pressure and 300 °C.^15,16^ Bifunctional materials, such as Ni-loaded 13X zeolite, have been mathematically modeled for the SEM process, showing that pure methane can be produced until the water breakthrough occurs.^17,18^ In addition, the same material has been studied under partial load and transient conditions demonstrating a high operational stability.^19^ Walspurger et al. validated the SEM process using a commercial Ni-based catalyst and zeolite 4A as the adsorbent.^13^ CO_2_ conversion close to 100% was achieved at atmospheric pressure over a temperature range of 250–350 °C. Gómez et al. obtained high-purity CH_4_ using a commercial Rh-based catalyst and zeolite 4A at atmospheric pressure and 275 °C, employing a mixture of H_2_/CO_2_ feed gas.^20,21^ Recently, the SEM process has been scaled up using a Ni-based catalyst and zeolite 4A for a synthetic syngas feed (i.e., a mixture of H_2_, CO, and CO_2_), obtaining a 100% CH_4_ purity in the outlet gas.^22^

As reported in the literature, zeolites are generally proposed as sorbents for the SEM process;^12−15,20−25^ however, there are alternative sorbents relatively unexplored for this application. Apart from zeolites, only two different sorbents have been considered as H_2_O sorbents in the SEM process.^24,26^ Lanthanum-based sorbents were unattractive due to their limited availability along with their high price. However, CaO seems to be an appealing alternative as the sorbent because of its low price, nontoxicity, high availability, and favorable conditions for regeneration and reuse.^27^ In addition, CaO satisfies an essential requirement for the SEM process by possessing a remarkable hydration capacity. As presented in a previous work performed by the authors, in the range of interest for the SEM process, from 200 to 350 °C and atmospheric pressure, CaO exhibited a high H_2_O capture capacity (i.e., ∼0.3 gH_2_O/gCaO) when exposed to 20 vol % H_2_O.^28^ It was also shown that increasing the H_2_O concentration in the reaction atmosphere resulted in an increase in the reaction rate at 250 °C. CaO has already been proposed as a sorbent for the SEM process by some authors in the literature.^24,26^ Agirre et al. studied the process for CO_2_ using CaO as the sorbent and Ni-based catalyst under 15 bar and 290 °C in a lab-scale fixed-bed reactor.^24^ However, unsatisfactory results were obtained due to catalyst deactivation during the experiment. Coppola et al. tested CaO as the sorbent under SEM conditions (i.e., temperature range from 200 to 300 °C and a feed composition containing H_2_O and CO_2_), employing a lab-scale dual fluidized bed system.^26^ The experimental results showed that the presence of CO_2_ in the reaction atmosphere had a negative effect on the H_2_O capture capacity of CaO due to the carbonation reaction. In a recent work published by the authors, a decrease in the hydration capacity was observed as a result of the formation of a CaCO_3_ product layer that partially sealed the reaction surface and hindered the interaction between available CaO and steam. The hydration capacity decreased from 0.99 to 0.27 molH_2_O/molCaO using a feed composition of 20 vol % H_2_O and 20 vol % H_2_O-10 vol % CO_2_, respectively.^28^ Coppola et al. conducted a thermodynamic analysis of the SEM process using ASPEN plus software considering CaO and a Ni-based catalyst as functional materials.^29^ The model consisted of a dual interconnected fluidized bed under isothermal conditions at an atmospheric pressure. Therefore, the SEM process was considered in the first reactor, while the regeneration of the sorbent took place in the interconnected reactor. They analyzed the composition of the product gas by varying the feed gas composition (i.e., H_2_/CO/CO_2_ mixture), the methanation temperature (i.e., 200, 250, and 300 °C), and the recirculation of CaO between the reactors. The thermodynamic analysis confirmed the negative effect of the undesired carbonation reaction on the SEM performance. However, by modifying the feed gas composition (i.e., hydrogen lean gas feed), suitable conditions were found for direct injection of the SNG in the natural gas distribution infrastructure. Despite the undesirable carbonation reaction, CaO remains an interesting sorbent for the SEM process because the produced CaCO_3_ can be recovered as high-value products (i.e., additional CH_4_) when decomposed under appropriate conditions. In the literature, some authors have already demonstrated the feasibility of converting CaCO_3_ into a syngas mixture (i.e., H_2_ and CO mixture) by calcination under a H_2_ atmosphere at 700–800 °C.^30^

Based on this context, the main objective of this study is to explore the potential of the SEM process using CaO as the sorbent in a scenario of biomass conversion into SNG, i.e., as a conversion stage of the syngas produced through biomass gasification. In this sense, hydrogenation of both CO and CO_2_ will occur. The approach followed in this study has been to perform a parametric study of the operating conditions of the SEM process under a CO_2_-free atmosphere and then to explore the presence of CO_2_ in the feed gas mixture fed to the process. The functional materials used included CaO as a sorbent and a commercial Ni-based catalyst. The operating parameters studied included the reaction temperature, H_2_/CO ratio in the feed gas, CO space velocity, and sorbent/catalyst mass ratio. Once the optimum operating conditions were determined, the stability of the materials was evaluated over 14 cycles. The CaCO_3_ generated during the SEM process was converted to CH_4_ during the regeneration stage using H_2_. Finally, the SEM performance was analyzed for feed gas mixtures containing H_2_/CO/CO_2_.

## 2 Experimental Section

### 2.1 Materials

The SEM process requires two functional materials: a methanation catalyst and a H_2_O sorbent. In this work, a natural CaO-based sorbent and a commercial Ni-based catalyst were considered, both in a size range of 100–200 μm. CaO was obtained by the calcination of a high-purity limestone (>92% CaO in calcined solid) at 850 °C for 60 min at atmospheric pressure in a muffle furnace. To evaluate the performance of the SEM process using a CaO sorbent with different BET surface areas, a sintered CaO-based sorbent was prepared. The calcination conditions used to produce sintered CaO included high calcination temperature and more reaction time (i.e., 1000 °C and 150 min, respectively). The textural characterization of the CaO-based sorbents used in this work is presented in Table 1. The CaO sorbent exhibited the typical values of BET surface area, pore size distribution, and density for CaO obtained from natural sources.^31^ While the sintered CaO presented textural characterization values comparable to those of a highly cycled CaO-based sorbent,^31^ the BET surface area was calculated by N_2_ adsorption (Micromeritics ASAP 2020) at 77 K using the Brunauer–Emmett–Teller equation. The pore diameter was estimated using a Hg porosimeter, Quantachrome Pore Master. The true density of both materials was determined by the helium picnometry technique using a Micromeritics AccuPyc II pycnometer. For the analysis of the solids used, sorbent and catalyst particles were manually separated using the magnetic properties of Ni and analyzed separately.

**Table 1:** Textural Characterization of CaO Utilized in This Work

	BET surface area (m^2^/g)	pore diameter (nm)	density (g/cm^3^)
CaO	16.8	61.9	3.19
Sintered CaO	5.7	191.0	3.24

The commercial Ni-based catalyst was supplied by Haldor Topsoe. The catalyst contains less than 20% Ni by weight. First, its catalytic activity was evaluated under conventional CO methanation conditions (i.e., without sorbent). This study was performed by mixing the catalyst with SiC (VWR Chemicals BDH Prolabo) in a catalyst/SiC mass ratio of 1:3. The particle sizes of the SiC and the catalyst were in the ranges of 200–300 and 100–200 μm, respectively.

### 2.2 Microfixed Bed Reactor Facility

Conventional CO methanation and SEM experiments were performed in a laboratory-scale fixed-bed reactor connected to a quadrupole mass spectrometer (MS) (Omnistar, Pfeiffer Vacuum), as shown in Figure S1. This facility has been previously described elsewhere.^20^ A fixed-bed microreactor consisted of a 30 mm long stainless-steel tube with an inner diameter of 6.8 mm. The reaction gases involved in this study (i.e., H_2_, CO_2_, CO, and Ar) were controlled by mass flow controllers. The mass-to-charge ratios used to analyze the composition of the product gas were as follows: H_2_ (*m*/*z* = 2), CH_4_ (*m*/*z* = 15), H_2_O (*m*/*z* = 18), CO (*m*/*z* = 28), Ar (*m*/*z* = 40), and CO_2_ (*m*/*z* = 28 and 44). Prior to each experiment, a calibration mixture containing 2.5 vol % H_2_, 2.5 vol % CO_2_, 2.5 vol % CO, and 2.5 vol % CH_4_ in Ar was used to calibrate the gases in the MS. In order to prevent the water condensation inside the connecting pipes, external resistances heated at 250 °C were utilized. Ar was used to dilute the gases prior to MS analysis. However, this dilution occurs downstream of the reactor, and the concentrations reported in this work correspond to an Ar-free basis. All experiments were performed at atmospheric pressure.

### 2.3 Experimental Routine

A schematic representation of the experimental routine followed in this paper is shown in Figure S2. A conventional CO methanation study was performed to evaluate the catalytic activity of the commercial catalyst. Catalyst reduction was carried out using a mixture containing 13 vol % H_2_ in Ar (total gas flow 34.5 N mL/min) at 700 °C. The reactor was then cooled to the desired reaction temperature while using 100 vol % Ar (total gas flow 30 N mL/min). The reaction temperatures considered in this study ranged from 200 to 300 °C. The amount of catalyst used in this study was set at 496 mg. The space velocities used were 0.25, 0.43, and 0.80 kg_CO_/kg_cat_ h. The reaction atmosphere studied included different H_2_/CO ratios in the feed gas (i.e., 2.5, 3, and 3.5).

The SEM process was studied under the same conditions of temperature, space velocity, reaction atmosphere, and H_2_/CO ratio in the feed gas as those used in the conventional CO methanation study. The catalytic bed was composed of a physical mixture of CaO and catalyst. The amount of catalyst and CaO was set at 850 mg each for all experiments, except in the study of the mass ratio CaO/Cat. In the study of the mass ratio CaO/cat, the amount of catalyst (i.e., 535 mg) in the solid bed was maintained constant during this analysis, while the amount of CaO was adjusted in order to obtain the desired CaO/catalyst ratio. Such an amount of catalyst was chosen to ensure that the solid bed remained within the isothermal zone of the reactor in all the experiments. During the SEM experiments, an additional stage was introduced to dehydrate Ca(OH)_2_ and to calcine CaCO_3_ formed during the reaction. The Ca(OH)_2_ dehydration was carried out at 450 °C in 100 vol % Ar, whereas calcination of CaCO_3_ was performed at 450 °C in 100 vol % H_2_. The presence of H_2_ in the reaction atmosphere promotes the methanation reaction of the CO_2_ released by the CaCO_3_ calcination, as already demonstrated by other authors in the literature.^32,33^ Jo et al. have already demonstrated that CO_2_ captured as CaCO_3_ by a Ni/CaO dual function (catalyst and sorbent) material produces CH_4_ at 500 °C.^32^ In this work, the hydrogenation of CaCO_3_ at moderate temperature has been employed as a method to convert the CaCO_3_ produced during SEM into CaO.

A cycle stability study of the materials for the SEM process was performed. Prior to the first cycle, catalyst reduction was performed at 700 °C with 13 vol % H_2_/Ar. Then, the SEM experiment was performed at 275 °C, with a stoichiometric H_2_/CO module as the feed gas, 0.8 kg_CO_/kg_cat_ h, and a CaO/cat mass ratio of 1. The test duration was maintained for ∼30 min until the composition of the post-breakthrough product gas reached that of conventional methanation. In this section, the Ca(OH)_2_ dehydration and CaCO_3_ calcination were performed in a single step at 450 °C in 100 vol % H_2_, denominated regeneration step. The SEM and the regeneration step were performed iteratively over 14 cycles. To verify the complete calcination of the formed CaCO_3_, the temperature of the regeneration step was achieved at 700 °C in cycles 1, 2, and 11.

The effect of feed gas mixtures containing CO_2_ was studied for the SEM process. The experiment started with the catalyst reduction at 700 °C under a 13 vol %H_2_/Ar atmosphere. After catalyst reduction, the reactor was cooled at a reaction temperature (i.e., 275 °C) under 100% Ar. The SEM experiment was then performed using a mixture containing 60 vol % H_2_, 19 vol % CO, and 21 vol % CO_2_. Finally, the regeneration step was performed at 700 °C under a H_2_ atmosphere to dehydrate Ca(OH)_2_ and calcine CaCO_3_.

The activity results were expressed in terms of CO conversion, H_2_ conversion, CH_4_ yield, CH_4_ purity in the outlet gas, CH_4_ selectivity, CaO hydration conversion, and CaO carbonation conversion. The equations utilized to describe the parameters are listed in Table 2. The species molar flow rate (*N*) is given in mol/min. The CaO hydration conversion was calculated by quantifying the H_2_O released by Ca(OH)_2_ dehydration. The CaO carbonation conversion was calculated according to CaCO_3_ calcination (eq 3) and CO_2_ methanation (eq 2):
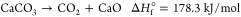
3

**Table 2:** Definition of the Parameters Used in the Discussion of the Results in This Work

parameter	equation	
CO conversion (*X*_CO_)	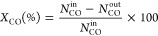	4
H_2_ conversion (*X*_H_2__)	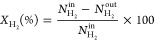	5
CH_4_ yield (η_CH_4__)	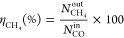	6
CH_4_ selectivity (*S*_CH_4__)	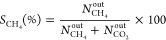	7
CaO hydration conversion (*X*_Ca(OH)**_2_**_)	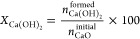	8
CaO carbonation conversion (*X*_CaCO_3__)	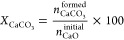	9

The CO_2_ released from CaCO_3_ reacts with H_2_ through a CO_2_ methanation reaction to produce CH_4_ and H_2_O. Therefore, the CaO carbonation conversion can be calculated through the quantification of the CH_4_ produced.

## 3 Results and Discussion

### 3.1 Catalytic Performance in Conventional CO Methanation

**Figure 1:**
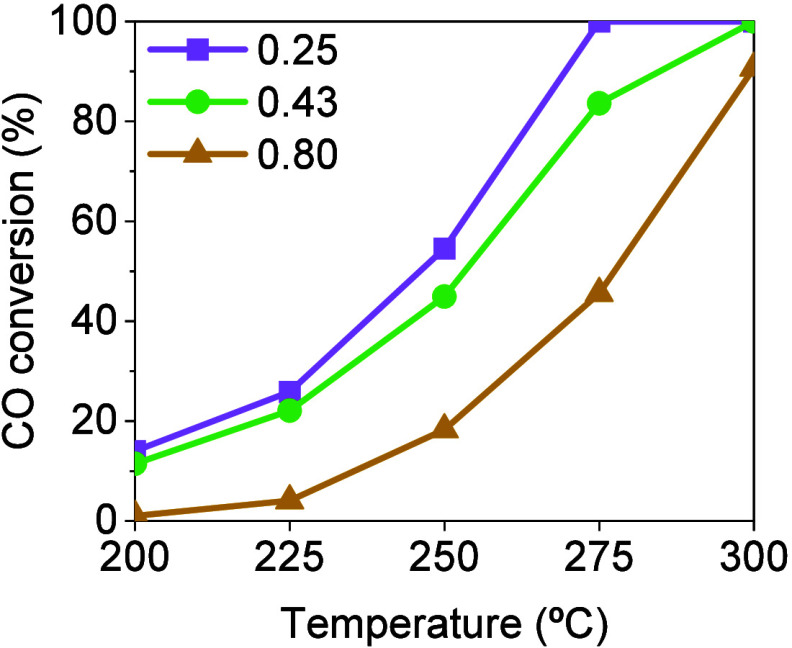
CO conversion obtained in conventional CO methanation at different space velocities (i.e., 0.25, 0.43, and 0.8 kg_CO_/kg_cat_ h) in a temperature range from 200 to 300 °C. Inlet gas composition: Stoichiometric H_2_/CO module with respect to CO methanation.

First, the catalytic activity of the commercial Ni-based catalyst for the CO methanation reaction was investigated. The temperature studied ranged from 200 to 300 °C since the CaO sorbents have already shown a noticeable H_2_O sorption capacity under such conditions in a previous study.^28^ In this work, three different space velocities (with respect to the mass of the catalyst) were considered: 0.25, 0.43, and 0.80 kg_CO_/kg_cat_ h. Figure 1 shows the experimental results for CO conversion at the different space velocities and reaction temperatures considered. As expected, the catalytic activity decreased with increasing space velocity at all temperatures studied. Then, at 275 °C, complete conversion was achieved at 0.25 kg_CO_/kg_cat_ h, while at 0.80 kg/kg_cat_ h, a maximum of 45.6% conversion was reached. According to the equilibrium thermodynamics, the methanation reaction is favored at low temperatures, as evidenced by the complete equilibrium CO conversion predicted at all temperatures considered (see Table S1). Nevertheless, the methanation reaction kinetics are slow, especially in the temperature range of interest for the SEM process, thus requiring the use of an active catalyst.^10^Table S2 shows the experimental results of the catalytic activity of the commercial catalyst in terms of *X*_CO_, *X*_H_2__, η_CH_4__, and CH_4_ purity in the outlet gas at different temperatures and space velocities.

In each experiment, according to the equilibrium calculations, CO_2_ was detected in the product gas composition, as it was produced by the WGS reaction (according to eq 10):
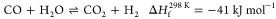
10

The effect of the H_2_/CO module in the feed gas was analyzed under a space velocity of 0.8 kg_CO_/kg_cat_ h at 275 °C. H_2_/CO modules in the feed gas of 2.5, 3, and 3.5 were considered. Table 3 shows the experimental results obtained under the operating conditions studied. The best performance in terms of CO conversion, CH_4_ yield, and CH_4_ concentration in the product gas was obtained using the stoichiometric H_2_/CO module with respect to the CO methanation reaction. When the H_2_/CO module was reduced, the CH_4_ yield decreased by 10% compared to the H_2_/CO modules of 3 and 3.5 due to a less than stoichiometric H_2_ supply. When the H_2_/CO module increased, a decrease in the CH_4_ concentration in the product gas was observed as a result of the excess of H_2_ in the feed gas.

**Table 3:** Catalyst Activity Results Obtained at Different H_2_/CO Modules in the Feed Gas under 275 °C and 0.8 kg_CO_/kg_cat_h

H_2_/CO	*X*_CO_ (%)	*X*_H_2__ (%)	η_CH_4__ (%)	*S*_CH_4__ (%)	[H_2_] (%)	[CH_4_] (%)	[H_2_O] (%)	[CO_2_] (%)	[CO] (%)
2.5	40.93	49.13	30.99	96.44	48.57	13.04	13.04	0.48	24.86
3	45.58	44.06	41.21	96.11	47.89	15.51	15.51	0.62	20.47
3.5	44.36	38.45	39.48	96.26	59.55	12.18	12.18	0.47	15.61

### 3.2 Parametric Study of the Operating Conditions for the SEM Process

**Figure 2:**
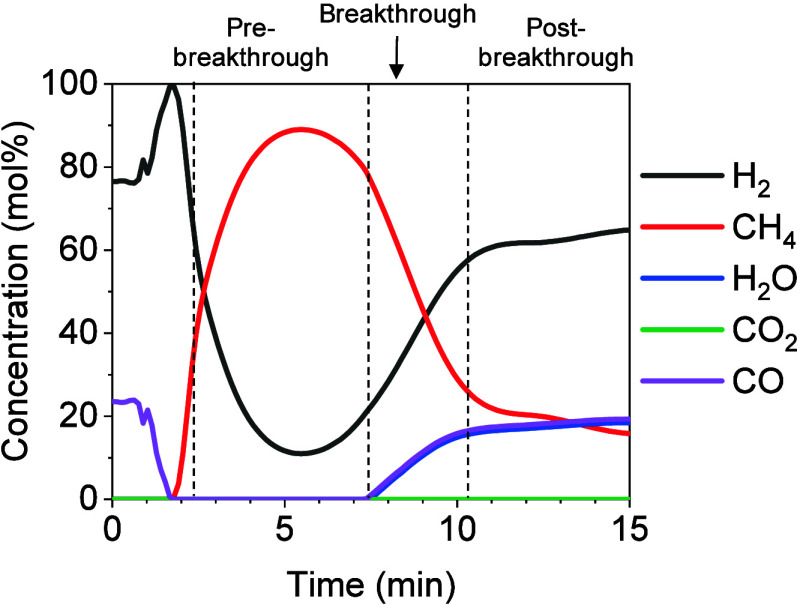
Concentration profile obtained in the SEM experiment at 275 °C and 0.8 kg_CO_/kg_cat_ h, atmospheric pressure, using a mixture of H_2_/CO in the ratio of 3:1 as feed and a CaO/cat mass ratio of 1.

In the previous section, the commercial Ni-based catalyst showed a significant activity for CO methanation within the temperature range of 200–300 °C at atmospheric pressure under different CO space velocities and H_2_/CO modules in the feed gas. In the same temperature range, CaO exhibited a significant hydration capacity, as reported in a recent study published by the authors.^28^ Consequently, the operating window for both functional materials, catalyst and H_2_O sorbent, was established in the temperature range of 200–350 °C and atmospheric pressure. Then, the parametric study of the operating conditions (i.e., temperature, H_2_/CO module, space velocity, and CaO/cat mass ratio) for sorption-enhanced CO methanation using both functional materials was carried out. The gas concentration profile obtained in the SEM process changes over time, as shown in Figure 2. At the beginning of the SEM, there is a pre-breakthrough period, where the sorbent is effectively removing the H_2_O formed through the methanation reaction and the reactions are therefore being displaced toward CH_4_ production. As a result, the content of CH_4_ fulfilled in this period is maximized and no H_2_O is detected in the product gas. Once the sorbent approaches saturation, there is a transient or breakthrough period, in which CH_4_ production decreases and H_2_, CO/CO_2_, and H_2_O appear in the product gas. Finally, when the sorbent is no longer active, a pure methanation gas composition is reached during the post-breakthrough period. In the following sections, when indicating single values for CH_4_ content during the SEM process, they correspond to the maximum values obtained experimentally in the pre-breakthrough stage. In a continuous process to produce SNG through the SEM process, the CH_4_ production stage would be completed immediately prior to the breakthrough, at which point the regeneration stage would begin. Thus, to operate the process with a continuous flow of SNG, a number of reactors operating in parallel will be needed.

#### 3.2.1 Effect of Temperature

As mentioned above, the individual evaluation of the catalyst and CaO-based sorbent served to establish the operating window in terms of the temperature for the SEM process. The effect of temperature in the SEM process was analyzed in the temperature range from 250 to 300 °C. Temperatures below 250 °C were not considered due to the poor catalyst performance in terms of CO conversion (less than 5%). Similarly, temperatures above 300 °C were not considered since 90% CO conversion was achieved in conventional methanation. As presented in a previous work performed by the authors, CaO showed a significant hydration capacity in the temperature range from 200 to 350 °C.^28^ The sorbent exhibited a hydration capacity of 0.3 mg H_2_O/mg CaO under 20 vol % H_2_O at atmospheric pressure in the temperature range mentioned above. Therefore, the effect of temperature on the SEM process was evaluated at 250, 275, and 300 °C using a stoichiometric H_2_/CO module with respect to the CO methanation reaction as feed, 0.8 kg_CO_/kg_cat_ h, and a CaO/cat mass ratio of 1.

Table 4 shows a comparison between the results obtained in conventional CO methanation (i.e., catalyst) and sorption-enhanced CO methanation (i.e., catalyst and sorbent) under identical operating conditions. A significant enhancement in the evaluated parameters was observed when the sorbent was introduced into the catalytic bed at 250 and 275 °C. In particular, at 275 °C, the maximum CO conversion increased from 45.6% in conventional CO methanation to 100% in SEM. At the same temperature, the maximum CH_4_ purity in the output stream was significantly improved from 15.5% as initial CH_4_ purity in conventional CO methanation up to ∼90% in SEM. By increasing the temperature up to 300 °C, almost no improvement in CO conversion was observed since a high conversion (i.e., 90.7% CO conversion) was already achieved in conventional CO methanation. Similarly, in the SEM experiment, a slight decrease in CH_4_ yield (i.e., from 86.7 to 81.1%) was observed when increasing the temperature from 275 to 300 °C. This decrease in the CH_4_ yield was attributed to the predominance of the WGS reaction at 300 °C in conventional CO methanation, resulting in a higher CO_2_ concentration in the outlet gas. As shown in Table S1, the equilibrium CO_2_ concentration in the outlet gas in conventional CO methanation increased from 1.16 to 1.60% when the temperature was increased from 275 to 300 °C due to the WGS reaction. The higher CO_2_ concentration in the environment combined with the higher temperature promoted the carbonation of CaO, as shown in a previous study.^28^ In this work, the conversion of CaO to CaCO_3_ was 2.03 and 5.60% at 275 and 300 °C, respectively, under identical operating conditions. This confirms the reduction of the CH_4_ yield due to the enhancement of the CaO carbonation reaction.

**Table 4:** Experimental Results Obtained in Conventional CO Methanation and SEM (Maximum Achieved in the Pre-Breakthrough Stage) Using a Mass Ratio CaO/Cat = 1 under Identical Operational Conditions: 0.8 kg_CO_/kg_cat_h and Using a Stoichiometric Module H_2_/CO as Feed (i.e., H_2_/CO = 3)

	*X*_CO_(%)		*X*_H_2__ (%)		η_CH_4__ (%)		[CH_4_] (%)	
	conv.	SEM	conv.	SEM	conv.	SEM	conv.	SEM
250 °C	18.3	43.5	19.03	43.07	14.1	31.7	4.3	28.7
275 °C	45.6	100	48.66	86.70	41.2	86.7	15.5	89.8
300 °C	90.7	100	88.48	89.70	77.6	81.1	35.2	79.7

Considering the experimental results presented in Table 4, 275 °C was selected as the most appropriate temperature due to the significant improvements observed in SEM experiments compared to conventional CO methanation. This study showed that a considerable initial catalytic activity is essential to obtain satisfactory SEM performance. Figure 2 shows the gas concentration profile obtained at 275 °C. The fluctuations in the concentration profile observed during the first ∼2 min correspond to the redirection of the gases from the bypass line to the reactor. Subsequently, the pre-breakthrough stage started and the concentration profile in the outlet gas showed a maximum CH_4_ content, i.e., a CH_4_ purity close to 90%. Complete CO conversion and no H_2_O were observed in the product gas as the sorbent effectively removed H_2_O. Once the sorbent began to saturate between minutes 6.5 and 10, the CH_4_ concentration decreased, while H_2_O and CO started to be detected. At the post-breakthrough stage, the sorbent was completely saturated, and the concentration profile obtained was similar to that of conventional methanation (i.e., CH_4_ purity of 15.5%).

#### 3.2.2 Effect of Module H_2_/CO

**Figure 3:**
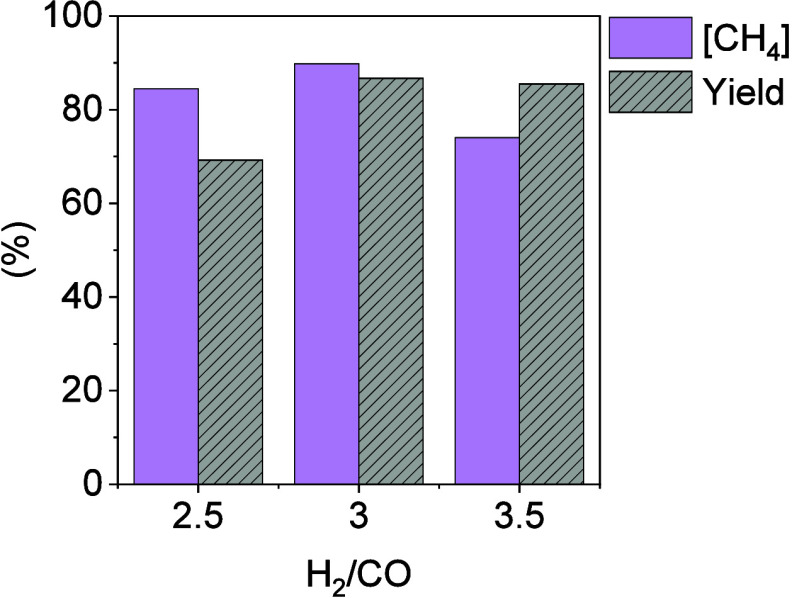
Results of maximum CH_4_ purity and yield obtained at the pre-breakthrough stage using different H_2_/CO feed gas ratios (i.e., 2.5, 3, and 3.5) at 275 °C, 0.8 kg_CO_/kg_Cat_ h, and mass ratio CaO/Cat = 1.

In order to maximize the catalytic performance of the SEM process, a study regarding the influence of the feed gas composition was investigated. Experiments were carried out with different H_2_/CO ratios in the feed gas (i.e., 2.5, 3, and 3.5) while keeping the operating conditions at 0.8 kg_CO_/kg_cat_ h, 275 °C, and a CaO/cat mass ratio of 1. CH_4_ purity and CH_4_ yield (shown in Figure 3) were the main parameters influenced by the variations in the H_2_/CO feed gas composition. As shown in Figure 3, the highest values of CH_4_ purity and CH_4_ yield were obtained using the stoichiometric module (i.e., H_2_/CO = 3) as feed. On the one hand, when the substoichiometric module (i.e., H_2_/CO = 2.5) was used as the feed gas, a significant reduction in CH_4_ yield was observed as a result of less than stoichiometric H_2_ supply. The CH_4_ yield decreased from 86.7 to 69.2% using the stoichiometric and substoichiometric modules, respectively. The CH_4_ purity in the outlet gas remained at similar values (i.e., 89.8 and 84.4%) using the stoichiometric and substoichiometric modules, respectively. On the other hand, using a H_2_/CO module higher than the stoichiometric (i.e., module H_2_/CO = 3.5), a considerable reduction in the CH_4_ purity due to the unreacted H_2_ was observed. The CH_4_ purity decreased from 89.8% for the stoichiometric module to 74.2% using the overstoichiometric module. The CH_4_ yield remained in similar values with respect to the stoichiometric module, being 86.7 and 85.5% using modules H_2_/CO of 3 and 3.5, respectively. The small differences observed in the CH_4_ yield were attributed to experimental error.

In all the experiments, complete CO conversion was observed during the pre-breakthrough stage. Both, the stoichiometric and overstoichiometric modules, supplied sufficient H_2_ to react with CO; hence, complete CO conversion was expected. However, when the substoichiometric module was used, a less than stoichiometric amount of H_2_ was supplied for the CO methanation reaction. Therefore, CO conversion was expected to be incomplete according to the reduction in CO conversion observed in conventional CO methanation using the substoichiometric module (see Table 3). However, in the SEM experiments, complete CO conversion was also observed as a result of the CaO carbonation reaction. Since the CO_2_ generated by the WGS reaction was consumed by the carbonation of CaO, the efficiency of the WGS reaction was increased. Consequently, unreacted CO was consumed by the WGS reaction, resulting in complete CO conversion. Therefore, the observed CaO conversion into a carbonate was slightly higher using the substoichiometric module under identical experimental conditions. Specifically, the conversion of CaO to CaCO_3_ was determined to be 1.67 and 1.50% using the substoichiometric and the stoichiometric modules, respectively.

#### 3.2.3 Effect of Space Velocity

Three different space velocities were considered in this study: 0.25, 0.43, and 0.8 kg_CO_/kg_cat_ h. The experimental conditions remained identical throughout the experiments (i.e., module H_2_/CO = 3 as the feed gas, 275 °C, and CaO/cat mass ratio = 1). Table 5 shows the experimental results obtained with the considered space velocities in conventional CO methanation and sorption-enhanced CO methanation. By using 0.25 kg_CO_/kg_cat_·h, an increase in the CH_4_ purity was observed from 37% in conventional CO methanation to 87.7% in sorption-enhanced CO methanation. However, the CO conversion and CH_4_ yield remained almost constant because high values were already obtained in conventional CO methanation (i.e., complete CO conversion and CH_4_ yield of 82.4%). The differences between conventional methanation and SEM became apparent when the CO conversion was incomplete. As the space velocity increased, the performance observed in conventional CO methanation decreased, as previously presented in Figure 1. Therefore, the improvement of the SEM process in terms of CO conversion, CH_4_ yield, and CH_4_ purity with respect to conventional CO methanation was noticeable. Using a space velocity of 0.43 kg_CO_/kg_cat_ h, the CO conversion increased from 83.6% in conventional CO methanation to 100% in SEM. Similarly, an increase in CH_4_ yield along with CH_4_ purity was observed. The CH_4_ yield was increased from 62.7% in conventional CO methanation to 89.5% in SEM. Similarly, the CH_4_ purity in SEM reached values of above 90%. At 0.80 kg_CO_/kg_cat_ h, the CO conversion obtained in SEM improved by 54.4% compared to that in conventional CO methanation. The CH_4_ yield value was doubled under SEM conditions compared to conventional CO methanation. Similarly, the CH_4_ purity was improved from 15.5% in conventional CO methanation up to 84.9% in SEM. As shown in Table 5, the best performance in terms of CH_4_ yield and CH_4_ purity, 89.5 and 93.2%, respectively, was reached, utilizing 0.43 kg_CO_/kg_cat_ h. However, these values were really close to those obtained at 0.80 kg_CO_/kg_cat_ h, where a lower initial catalytic activity was demonstrated. Therefore, by using higher space velocities, the improvements obtained in SEM were more noticeable than at lower space velocities.

**Table 5:** Comparison between Results Obtained Using Different Space Velocities in Conventional CO Methanation and SEM under the Same Operational Conditions (i.e., Stoichiometric Module H_2_/CO as Feed and 275 °C as Reaction Temperature)^a^

	*X*_CO_ (%)		*X*_H_2__ (%)		η_CH_4__ (%)		[CH_4_] (%)	
kg_CO_/kg_cat_ h	conv.	SEM	conv.	SEM	conv.	SEM	conv.	SEM
0.25	100	100	90.99	96.81	82.4	84.6	37.9	87.6
0.43	83.6	100	80.76	97.87	62.7	89.5	30.3	93.2
0.80	45.6	100	44.66	96.46	41.2	86.7	15.5	88.9

a In the SEM experiment, the mass ratio CaO/cat was set at 1. Note that the SEM values represented the maximum achieved in the pre-breakthrough stage.

The use of high space velocity can reduce secondary reactions such as the WGS reaction in the SEM process. The WGS reaction kinetics is slower compared to those of the CO methanation and CaO carbonation reactions. Experimentally, it was observed that using 0.80 kg_CO_/kg_cat_ h resulted in a 2.03% conversion of CaO to CaCO_3_. However, using 0.25 kg_CO_/kg_cat_ h, a higher conversion of CaO to CaCO_3_ was observed (i.e., 3.65%). Therefore, the use of high space velocity can limit the secondary reactions in SEM, promoting main reactions like methanation. In addition, for large-scale implementation of the process, working at higher space velocities offers further advantages such as higher productivity, reactor efficiency, and selectivity control.

#### 3.2.4 Effect of CaO/Cat Mass Ratio

**Figure 4:**
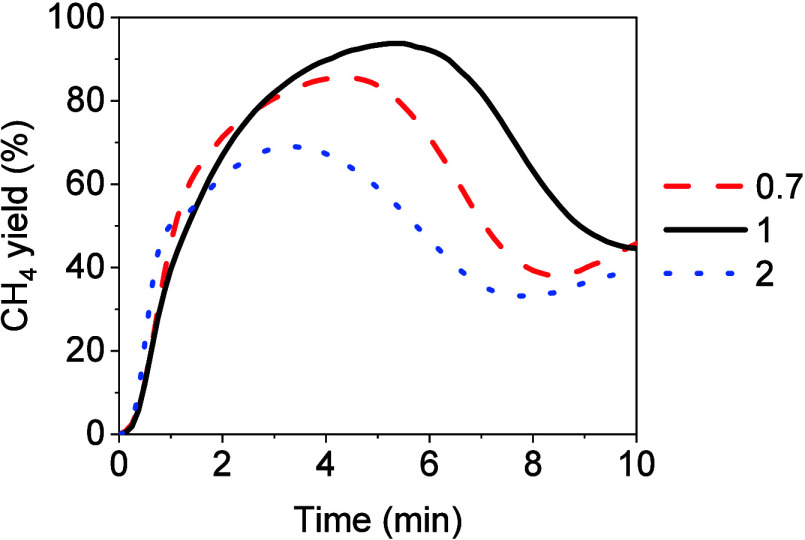
Yield to CH_4_ obtained utilizing different CaO/Cat mass ratios (i.e., 0.7, 1, and 2) under 275 °C, 0.8 kg_CO_/kg_cat_ h, and stoichiometric H_2_/CO as feed.

The SEM performance was evaluated with different CaO-to-catalyst mass ratios (i.e., CaO/cat of 0.7, 1, and 2). The experimental conditions were identical in all experiments: 0.8 kg_CO_/kg_cat_ h, 275 °C, and stoichiometric H_2_/CO module with respect to the CO methanation reaction as the feed gas. Figure 4 illustrates the results of the CH_4_ yield versus time obtained utilizing the different CaO/cat mass ratios in the pre-breakthrough stage. The first 2 min of the graph corresponded to the redirection of the gases from the bypass to the reactor. The maximum value of the CH_4_ yield was obtained with a mass ratio CaO/cat of 1. A decrease in the CH_4_ yield was observed when the CaO/cat mass ratio was decreased to 0.7. This decrease in CH_4_ yield became more pronounced as the CaO/cat mass ratio was increased to 2. In terms of CH_4_ purity in the product gas, the maximum value (i.e., CH_4_ concentration of 88.6%) was achieved utilizing the CaO/cat mass ratio of 1. By reducing the CaO/cat mass ratio to 0.7, a decrease in CH_4_ purity was noticed, reaching a CH_4_ concentration of 78.3% at the pre-breakthrough stage. A further reduction was observed utilizing a CaO/Cat mass ratio of 2, resulting in a CH_4_ concentration of 50.7% at the pre-breakthrough.

The experimental investigation showed that employing the CaO/cat mass ratio of 0.7, the amount of H_2_O removed from the gas phase was less than that removed using a CaO/cat mass ratio of 1. Specifically, 2.67 and 3.91 mmol of H_2_O were absorbed utilizing mass ratios of 0.7 and 1, respectively. Therefore, employing the CaO/cat mass ratio of 0.7 resulted in an increased presence of the H_2_O concentration in the gas phase. The presence of H_2_O along with the unreacted CO favored the WGS reaction producing CO_2_. Thus, the CaO carbonation reaction was favored, leading to a decrease in the overall performance of the process.

Employing a CaO/cat mass ratio of 2, a pronounced decrease in the SEM performance was observed at the pre-breakthrough stage. The larger amount of CaO used in this case, compared to the CaO/cat mass ratio of 1, enhanced the WGS reaction since the CO_2_ produced through this reaction was continuously removed from the gas phase due to CaO carbonation. Therefore, the amount of CO_2_ that reacted with CaO to produce CaCO_3_ was higher than that observed with a CaO/cat mass ratio of 1. The quantification of CO_2_ that reacted with CaO was determined experimentally by calcination at 450 °C under 100 vol % H_2_, considering the amount of CH_4_ produced in this stage, which corresponds to the hydrogenation of the CO_2_ released according to Section 2.3. Using a CaO/cat mass ratio of 2, the amount of CO_2_ reacted was determined to be 0.34 mmol of CO_2_. Using a CaO/cat mass ratio of 1, the amount of CO_2_ reacted was calculated to be 0.28 mmol. Considering the experimental results of this study, the best SEM performance was obtained by utilizing a CaO/cat mass ratio of 1.

### 3.3 Cycle Stability

To evaluate the stability of the functional materials and the reproducibility of their performance in the SEM process, a cycle stability test was carried out under fixed operating conditions. Specifically, 14 cycles of SEM and regeneration/reduction stages were performed according to the experimental procedure described in Section 2.3. The SEM stage was carried out at 275 °C, using a H_2_/CO module of 3 as the feed gas with a space velocity of 0.8 kg_CO_/kg_cat_ h and utilizing a CaO/cat mass ratio of 1. Between successive SEM cycles, the regeneration step was performed at 450 °C under a H_2_ atmosphere (30 N mL/min).

**Figure 5:**
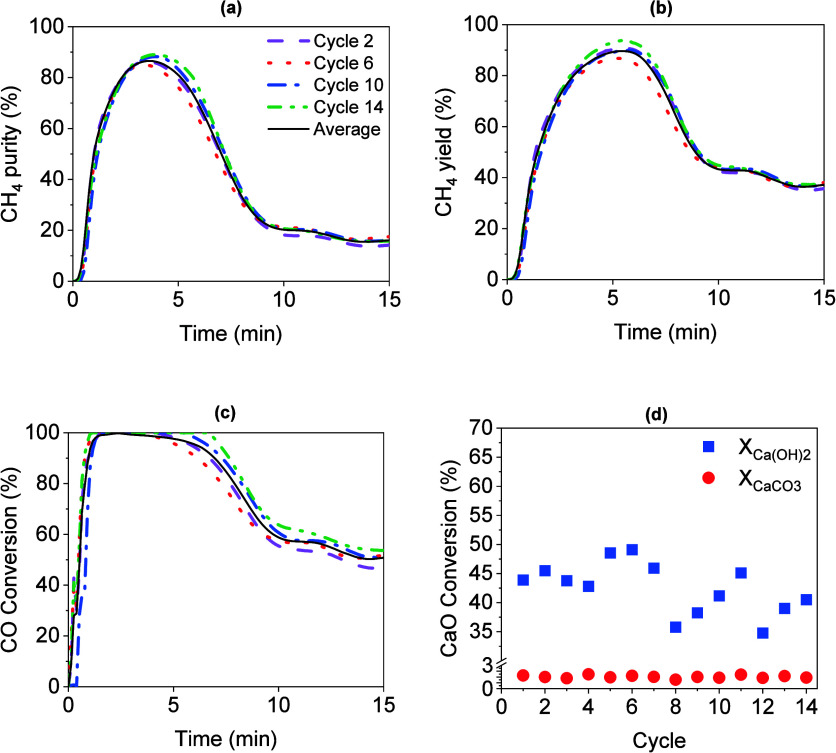
Results of CH_4_ purity (a), CH_4_ yield (b), CO conversion (c), and CaO conversion (d) obtained in the stability cycle test. Note that graphs (a), (b), and (c) share the same legend.

**Figure 6:**
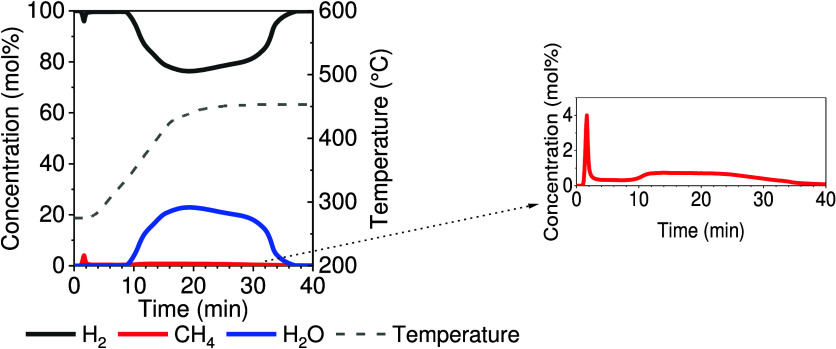
Concentration profile obtained in the cycle stability test during the regeneration stage utilizing a hydrogen atmosphere and increasing the temperature from 275 to 450 °C.

The experimental results in terms of CH_4_ purity, CH_4_ yield, CO conversion, and CaO conversion are reported in Figure 5. An average curve of the 14 cycles (represented by a solid line) and cycles 2, 6, 10, and 14 was introduced in the representation. Generally, good reproducibility with the cycles was observed despite the small differences due to the accuracy limits of the mass flow controllers. The first 2 min of Figure 5a–c corresponded to the redirection of the feed flow from the bypass to the reactor; therefore, the values started from zero and increased as the reaction progressed. The maximum values of CH_4_ purity, CH_4_ yield, and CO conversion obtained corresponded to the pre-breakthrough stage. Subsequently, the values started to decrease as the sorbent started to saturate in the breakthrough stage. The last 5 min of Figure 5a–c represents the post-breakthrough stage, where the sorbent was already fully saturated. The high reproducibility observed along the cycles in Figure 5 confirmed that the regeneration step was carried out satisfactorily. The average CaO conversion was calculated to be 42.44 and 2.03% in Ca(OH)_2_ and CaCO_3_, respectively. During the regeneration step, it was intended to dehydrate the Ca(OH)_2_ produced in the SEM, hydrogenate the CaCO_3_, and reduce the catalyst. Therefore, the composition of the outlet gas consisted mainly of H_2_O and CH_4_ in a H_2_ atmosphere, as shown in Figure 6. Predominantly, the obtained H_2_O was attributed to the dehydration of Ca(OH)_2_, while a small fraction was derived from the methanation reaction of the CO_2_ produced during CaCO_3_ calcination. The CH_4_ observed in the concentration profile resulted from the hydrogenation of CaCO_3_. The CO_2_ released from the carbonate reacts with H_2_ to produce CH_4_ and H_2_O. Therefore, the CH_4_ production was favored until CaCO_3_ produced during SEM was completely calcined.

It is well-known in the literature that cycling CaO can cause a reduction in BET surface area, resulting in a decrease in CaO conversion due to sintering phenomena.^34−37^ Wang et al. investigated the effect of successive calcination/carbonation cycles on the BET surface area of CaO. The calcination conditions were 900 °C in an atmosphere composed of 80% CO_2_ and 20% O_2_, followed by carbonation at 650 °C in a mixture of 15% CO_2_ and 85% N_2_. A reduction of 77.7 and 89.2% in the initial CaO BET surface area was determined in cycles 10 and 30, respectively.^38^ Martínez et al. noticed a reduction in the BET surface area of CaO from 17 m^2^/g (initial calcination) to 6 m^2^/g by the fifth calcination cycle. The cycles performed consisted of calcination in air at 900 °C for 5 min and carbonation under 10 kPa of CO_2_ in air at 650 °C for 5 min.^31^ The reaction conditions employed in this work are significantly different from those documented in the existing literature. In this study, the sorbent was texturally characterized after 14 cycles of SEM and regeneration step. At the end of the cyclic test, the material was recovered in the form of CaO, and no fragmentation was observed. The resulting BET surface area was determined to be 11.3 m^2^/g. Therefore, the sorbent experienced a 32.96% reduction in its initial BET surface area (16.8 m^2^/g). This reduction in BET surface area did not affect the CaO conversion, resulting in consistently reproducible results across cycles, as observed in Figure 5. The stability of the CaO conversion was attributed to the presence of steam in the reaction atmosphere, which may facilitate the continuous reactivation of the sorbent, a phenomenon that has been extensively documented in the literature.^31,39−41^ However, a progressive reduction in the BET surface area of CaO may occur if the cycling test continues indefinitely.

**Figure 7:**
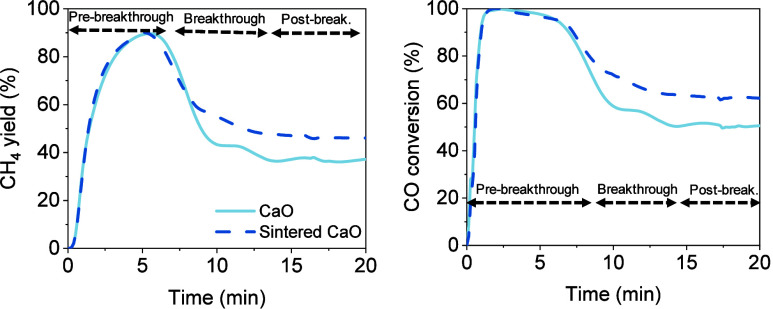
Comparison between the average cycle curve of CaO and sintered CaO in terms of the yield to CH_4_ (a) and CO conversion (b). Note that both graphs share the same legend.

To determine the effect that a reduced BET surface area might have on the SEM process, a stability study was conducted using a sintered CaO-based sorbent. To produce the sintered CaO, the original limestone was calcined under intensified conditions, as described in Section 2.1. The resulting BET surface area of the sintered CaO was determined to be 5.8 m^2^/g, almost 3 times smaller than that of the original CaO previously used (i.e., 16.9 m^2^/g). Similarly, 14 cycles of SEM and a regeneration step were carried out using sintered CaO under the identical cycle operational conditions described above. As previously performed, an average curve for the 14 cycles was calculated for sintered CaO. Figure 7 shows a comparison between the average cycle curve of the CH _4_ yield and the CO conversion for both materials. At the pre-breakthrough stage, a close similarity between the sorbents was observed, as the curves overlap each other for both CH _4_ yield and CO conversion. However, in the post-breakthrough, the sintered CaO showed up to 10% higher CH _4_ yield and CO conversion than the original CaO. On average, the CaO conversion was calculated to be 2.63 and 28.54% of the conversion to CaCO _3_ and Ca(OH)_2_, respectively. The CaO conversion obtained in each cycle is shown in Figure S3. A significant reactivation of the sorbent was observed during the first cycles. The initial conversion of CaO to Ca(OH)_2_ was determined to be 15.26%, and then, the conversion progressively increased to 27.87% in the third cycle and remained stable in the following cycles. The sintered CaO showed a reduced conversion to Ca(OH)_2_ compared to the original CaO. Specifically, the conversion of CaO to Ca(OH)_2_ achieved with sintered CaO corresponded to ∼70% of the capacity observed utilizing the original CaO. This is in strong agreement with that reported in a previous work published by the authors.^28^^28^

The hydration capacity of CaO is strongly influenced by the BET surface area. Therefore, the BET surface area of sintered CaO was determined after 14 cycles, resulting in 8.7 m^2^/g. An increase in the BET surface area was observed due to the reactivation of the sorbent by the hydration reaction. During the SEM process, Ca(OH)_2_ is formed and then dehydrated, resulting in an increase in the reactive surface area of sintered CaO. This phenomenon has been widely utilized by many authors in the literature to improve the performance of CaO-based sorbents. Martínez et al. noticed an increase in the BET surface area of CaO from 6 to 13 m^2^/g by steam reactivation.^31^ Dong et al. observed an increase in the BET surface area of CaO by steam reactivation at different H_2_O concentrations.^42^

A previous investigation conducted by the authors demonstrated that the hydration kinetics of sintered CaO was slower than that of the original CaO.^28^ The reactivation of sintered CaO along with the hydration reaction kinetics is essential to understanding the performance observed in the SEM process. The slow reaction kinetics can modify the catalytic activity in the SEM process because the CaO conversion rate can affect the methanation reaction rate. In heterogeneous catalysis, where the reaction takes place at the interface between a solid phase and a gas phase, slow reaction kinetics can facilitate the selective adsorption of the reactants and, thus, the formation of methane. As observed in Figure 7, calcined and sintered CaO exhibited similar performance during the pre-breakthrough stage of the SEM process as their curves overlap. This was attributed to the reactivation of the hydration capacity of the sintered material, as observed in the initial cycles shown in Figure S3. Since this stage corresponds to the period in which maximum CH_4_ purity is achieved, the results obtained corroborate the possibility of using highly cycled CaO-based sorbents in SEM as the reduced BET surface area does not impact the performance during this period. In an industrial-scale SEM process for producing SNG, the regeneration stage would start immediately after the pre-breakthrough stage; therefore, various reactors operating in parallel would be needed to ensure continuous SNG production. Consequently, the post-breakthrough stage would not be relevant to the overall process.

### 3.4 SEM Performance for H_2_/CO/CO_2_ Mixture as the Feed Gas

In the previous sections, the SEM process was studied considering CO methanation as the main reaction; hence, a feed gas containing exclusively H_2_ and CO was used. There, the CaO-based sorbent showed promising results in terms of CH_4_ yield, CO conversion, and CH_4_ purity. However, CO_2_ is typically present in most syngas obtained from biomass and waste gasification.^9,43,44^ Therefore, it was necessary to investigate the effectiveness of the SEM process when using a feed gas containing CO_2_. The composition of the feed gas used was 60 vol % H_2_, 19 vol % CO, and 21 vol % CO_2_. The operating conditions were set at 275 °C, a CaO/cat mass ratio of 1, a H_2_/CO module of 3.1, 0.8 kg_CO_/kg_cat_ h, and 1.2 kg_CO2_/kg_cat_ h. The catalytic bed used (i.e., catalyst and original CaO) was derived from the cycled stability test; therefore, it has previously undergone 14 cycles of SEM. A stoichiometric amount of H_2_ with respect to the CO methanation reaction was used because it was assumed that the CO_2_ present in the feed gas would react preferentially with CaO to produce CaCO_3_, as reported in the literature.^26^

**Figure 8:**
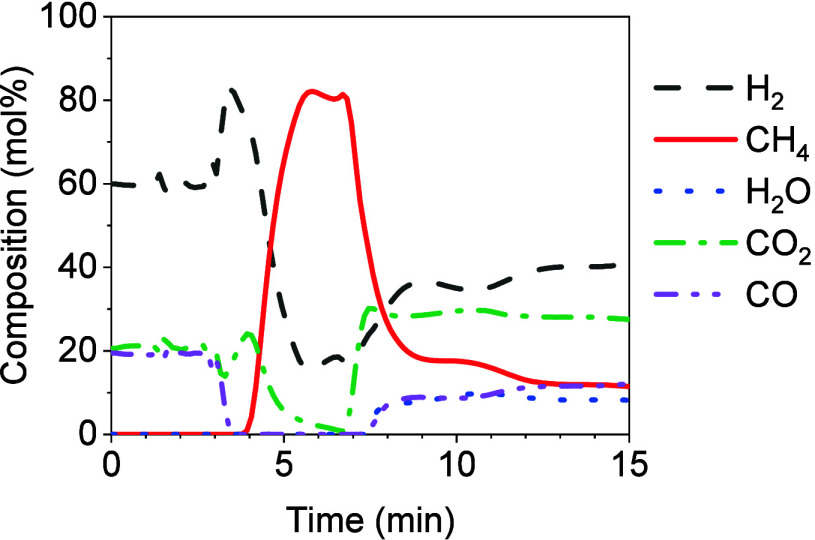
Outlet gas concentration profile using 0.8 kg_CO_/kg_cat_ h and 1.2 kg_CO2_/kg_cat_ h at 275 °C, CaO/cat = 1, and a feed gas composed by 60 vol % H_2_, 19 vol % CO, and 21 vol % CO_2_.

Figure 8 shows the concentration profile obtained at the reactor outlet. The first 4 min of the concentration profile corresponded to the stabilization of the reactive gases in the bypass line. Subsequently, some fluctuations were observed due to the redirection of the gases from the bypass to the reactor. During the pre-breakthrough stage, a maximum CH_4_ concentration of ∼82% was reached. The duration of the pre-breakthrough was reduced compared to the previous section, where no CO_2_ was considered under the same operating conditions (see Figure 2). The presence of CO_2_ in the feed gas resulted in a reduction in the available CaO for the hydration reaction as the carbonation and hydration reactions occurred simultaneously. The conversion of CaO to CaCO_3_ and Ca(OH)_2_ was calculated, as described in Section 2.3. The conversion of CaO resulted in a 9.20% conversion to CaCO_3_ and an 8.90% conversion to Ca(OH)_2_, respectively. It was observed that the use of a H_2_/CO/CO_2_ mixture as a feed gas resulted in a lower conversion of CaO to Ca(OH)_2_ compared to that using only H_2_/CO as a feed gas, where the conversion of CaO to Ca(OH)_2_ was determined to be 42.44%. This reduction was attributed to the formation of CaCO _3_ on the surface of the particles, which prevented the CaO from interacting with H_2_O.^28^ As shown in Figure 8, the breakthrough stage corresponded to the saturation of the sorbent, and then, H_2_O started to be detected in the MS. In the post-breakthrough stage, an increase in the CO_2_ concentration with respect to the initial CO_2_ present in the feed gas was observed and it was attributed to the WGS reaction. As observed in Figure 8, the composition of the outlet gas in the pre-breakthrough stage was approximately 82 vol % CH_4_ and 18 vol % H_2_, similar to that of hytane gas. Hythane is defined by a mixture of 10–25 vol % H_2_ and 90–75 vol % CH_4_ suitable for its application as a transportation fuel.^45^ Hytane resulted in an interesting alternative in the transportation fuel sector as it can promote the transition from fossil fuels to renewable energy.

As mentioned above, CO_2_ is typically present in different concentrations in most syngas samples, regardless of the origin. The reaction between CO_2_ and CaO has a negative effect on the duration of the SEM and on the overall process yield. In this work, the regeneration of CaCO_3_ produced in SEM was carried out by the hydrogenation reaction producing CH_4_ and H_2_O. In an industrial-scale process, the required H_2_ would be by means of water electrolysis using renewable energy, and the product gas obtained could be recirculated to the SEM process to adjust the required H_2_/CO feed module.

## 4 Conclusions

In this work, a parametric study of the sorption-enhanced CO methanation process using CaO as a sorbent and a commercial Ni-based catalyst has been carried out. The performance of both materials in the SEM process was evaluated at different temperatures, H_2_/CO module in the feed gas, CO space velocity, and CaO/cat mass ratio. High-purity methane (∼90% CH_4_ concentration) was obtained in the outlet gas at 275 °C, with module H_2_/CO = 3 as the feed gas with a space velocity of 0.8 kg_CO_/kg_cat_ h and a mass ratio CaO/cat of 1. Moreover, stable performance of the materials was observed under SEM conditions over the 14 cycles. The effect of the BET surface area in a CaO-based sorbent for the SEM process was investigated using a sintered CaO. It was found that the reduced BET surface area of the sintered material did not affect the pre-breakthrough stage of the process. Therefore, a highly cycled CaO sorbent derived from other chemical processes such as the calcium looping process could be reused as a sorbent for the SEM process. Finally, an experiment was conducted using a feed gas similar to that obtained from biomass gasification (i.e., a mixture consisting of 60% H_2_, 19% CO, and 21% CO_2_). It was found that the CaO carbonation reaction resulted in a decrease in the pre-breakthrough time of the process. Still, it is interesting to highlight that the gas composition obtained, approximately 82% vol CH_4_ and 18% vol H_2_, can be considered suitable for its use as a hythane gas and so be used as fuel in the transport sector, contributing to the decarbonization of such sector. At the same time, carbonate regeneration with H_2_ has been demonstrated to effectively produce CaO, which can be reused cyclically, along with CH_4_.

## Supporting Information Available

Supplementary equilibrium calculations and additional experimental information (PDF)
