# Supplementary material for: Sorption-Enhanced Methanation Using CaO- and Ni-Based Catalysts as Functional Materials
Source: Energy Fuels. 2025 Feb 25;39(9):4304–14. doi: 10.1021/acs.energyfuels.4c04828 (PMC11891897; doi:10.1021/acs.energyfuels.4c04828)
Supplement: Supplementary file 1 — ef4c04828_si_001.pdf [file ef4c04828_si_001.pdf]

SUPPORTING INFORMATION

Sorption Enhanced Methanation (SEM) using CaO and Ni-based catalyst as functional materials

*Yusbeli C. García, Gemma Grasa, Isabel Martínez**

Environmental Research Group, Instituto de Carboquímica (Spanish National Research Council, ICB-CSIC), Miguel Luesma Castán 4, 50018 Zaragoza (Spain)

*Corresponding author: imartinez@icb.csic.es

Table S1. Equilibrium calculations based on minimization of Gibbs free energy using a module H_2/CO of 3 as feed gas under different temperatures and atmospheric pressure. The e calculations were obtained using the commercial software Aspen Plus V12.1.

**Temp. (ºC)**	X_{CO} (%)	X_{H_2} (%)	η_{CH_4} (%)	S_{CH_4} (%)	$[H_2]$ (%)	$[CH_4]$ (%)	$[H_2O]$ (%)	$[CO_2]$ (%)	$[CO]$ (%)
**200**	100	99.05	98.58	99.29	1.41	49.30	48.94	0.35	0.00
**225**	100	98.53	97.80	98.90	2.18	48.91	48.36	0.54	0.00
**250**	100	97.81	96.71	98.36	3.24	48.38	47.57	0.81	0.00
**275**	100	96.84	95.26	97.63	4.63	47.68	46.53	1.16	0.00
**300**	99.99	95.59	93.39	96.70	6.40	46.80	45.20	1.60	0.01

Table S2. Catalytic activity of the commercial Ni-based catalyst for CO methanation reaction at different temperatures and CO spatial velocities.

Temp. (ºC)	Vesp ($kg_{CO}/kg_{cat} \cdot h$)	X_{CO}(%)	X_{H_2}(%)	η_{CH_4}(%)	$[CH_4]$(%)
**200**	0.25	14.01	13.02	1.72	0.50
	0.43	11.45	11.16	1.11	0.31
	0.80	1.03	1.01	0.64	0.16
**225**	0.25	25.85	23.32	8.87	2.92
	0.43	22.11	18.16	5.88	1.76
	0.80	4.07	3.75	3.37	0.87
**250**	0.25	54.56	50.96	34.07	14.67
	0.43	44.93	41.96	25.29	9.32
	0.80	18.27	17.56	14.05	4.24
**275**	0.25	100	91.01	82.43	37.94
	0.43	83.61	80.84	62.67	30.32
	0.80	45.58	44.59	41.21	15.51
**300**	0.25	100	96.85	92.06	42.57
	0.43	100	96.02	86.54	43.00
	0.80	90.69	87.48	77.55	35.22

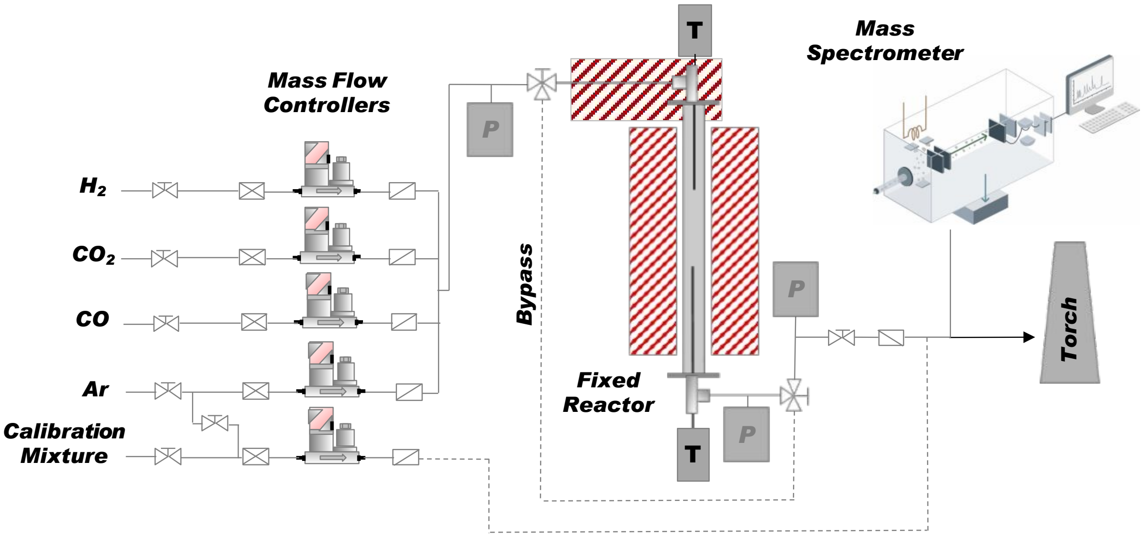

Figure S1. Laboratory-scale fixed micro-reactor connected to a MS used in this work.

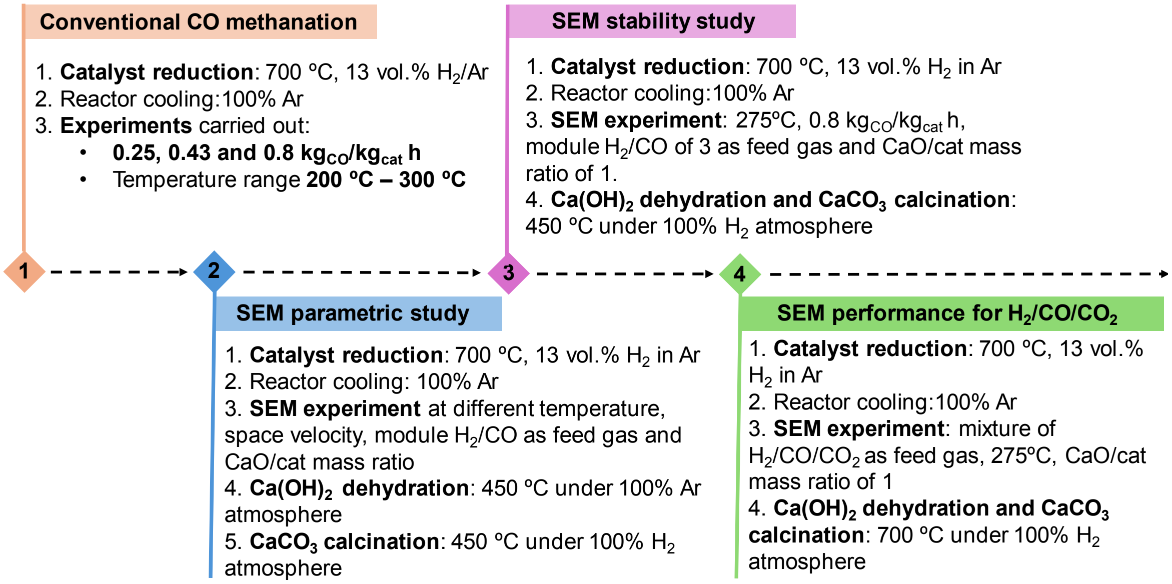

Figure S2. Schematic representation of the sequence of the experiments carried out.

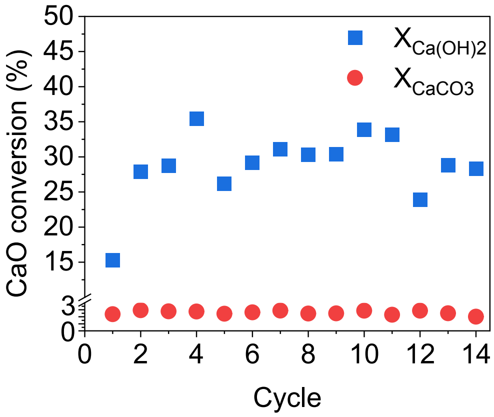

Figure S3. CaO conversion into $Ca(OH)_2$ and $CaCO_3$ obtained utilizing sintered CaO. Operational conditions: $0.8kg_{CO}/kg_{cat}$ h, 275 ºC, $H_2/CO=3$ as feed gas and mass ratio CaO/cat=1.
